# Prion-mediated neurodegeneration is associated with early impairment of the ubiquitin–proteasome system

**DOI:** 10.1007/s00401-015-1508-y

**Published:** 2015-12-08

**Authors:** Chris McKinnon, Rob Goold, Ralph Andre, Anny Devoy, Zaira Ortega, Julie Moonga, Jacqueline M. Linehan, Sebastian Brandner, José J. Lucas, John Collinge, Sarah J. Tabrizi

**Affiliations:** Department of Neurodegenerative Disease, University College London, Institute of Neurology, Queen Square, London, WC1N 3BG UK; Centro de Biología Molecular “Severo Ochoa”, (CBMSO) CSIC/UAM, Madrid, Spain; Networking Research Center on Neurodegenerative Diseases (CIBERNED), Instituto de Salud Carlos III, Madrid, Spain; MRC Prion Unit, University College London, Institute of Neurology, Queen Square, London, UK; Division of Neuropathology, The National Hospital for Neurology and Neurosurgery, Queen Square, London, UK

**Keywords:** Prion, PrP, UPS, Proteasome, Neurodegeneration

## Abstract

Prion diseases are a group of fatal neurodegenerative disorders characterised by the accumulation of misfolded prion protein (PrP^Sc^) in the brain. The critical relationship between aberrant protein misfolding and neurotoxicity currently remains unclear. The accumulation of aggregation-prone proteins has been linked to impairment of the ubiquitin–proteasome system (UPS) in a variety of neurodegenerative disorders, including Alzheimer’s, Parkinson’s and Huntington’s diseases. As the principal route for protein degradation in mammalian cells, this could have profound detrimental effects on neuronal function and survival. Here, we determine the temporal onset of UPS dysfunction in prion-infected Ub^G76V^-GFP reporter mice, which express a ubiquitin fusion proteasome substrate to measure in vivo UPS activity. We show that the onset of UPS dysfunction correlates closely with PrP^Sc^ deposition, preceding earliest behavioural deficits and neuronal loss. UPS impairment was accompanied by accumulation of polyubiquitinated substrates and found to affect both neuronal and astrocytic cell populations. In prion-infected CAD5 cells, we demonstrate that activation of the UPS by the small molecule inhibitor IU1 is sufficient to induce clearance of polyubiquitinated substrates and reduce misfolded PrP^Sc^ load. Taken together, these results identify the UPS as a possible early mediator of prion pathogenesis and promising target for development of future therapeutics.

**Electronic supplementary material:**

The online version of this article (doi:10.1007/s00401-015-1508-y) contains supplementary material, which is available to authorized users.

## Introduction

Impairment of the ubiquitin–proteasome system (UPS) has been implicated in a variety of neurodegenerative disorders including Alzheimer’s, Parkinson’s, Huntington’s and prion diseases. The UPS is a highly conserved and tightly regulated pathway in which covalent conjugation of a polyubiquitin chain to redundant or misfolded proteins leads to rapid degradation by the 26S proteasome [9]. Despite heterogeneity in clinical presentation, different neurodegenerative disorders are characterised by the accumulation of ubiquitinated conjugates in post-mortem brain tissue [31]. In the case of certain human prion diseases, ubiquitin is deposited as coarse granules at the periphery of prion amyloid plaques, which are composed of disease-associated PrP^Sc^, a misfolded and aggregation-prone conformer of the normal cellular protein, PrP^C^ [20, 37]. These observations gave rise to the hypothesis that UPS impairment may contribute to neurotoxicity in prion diseases through a progressive loss of cellular proteostasis.

A number of studies in cellular and animal models provide evidence to suggest that UPS dysfunction may be important in prion disease pathogenesis. Mice infected with the ME7 prion strain show impairment of proteasome chymotrypsin-like and caspase-like activities and increased levels of ubiquitinated conjugates [22]. We replicated these findings in the brains of wild-type mice infected with the RML prion strain, confirming that proteasome impairment is a general feature of prion disease pathogenesis, rather than specific to a single prion strain [24]. Co-immunoprecipitation experiments demonstrated a direct interaction between PrP^Sc^ and components of the 26S proteasome in brains of prion-infected mice [7]. In follow-up experiments, we identified a novel mechanism of proteasome impairment in which aggregated β-sheet-rich PrP inhibits the proteasome by stabilising the closed conformation of the substrate entry channel [7]. Consistent with a loss of proteasome activity, levels of the short-lived endogenous proteasomal substrates p27, p53 and IĸBα are elevated in the brains of mice infected with the RML prion strain [7]. Aberrant accumulation of such critical short-lived proteins could be a key source of neurotoxicity in prion disease pathogenesis.

Various transgenic UPS reporter mouse lines have been developed to measure the functional status of the UPS in vivo. Ub^G76V^-GFP transgenic mice express a reporter protein with a ubiquitin fusion degradation (UFD) signal, consisting of an N-terminally linked ubiquitin moiety for accepting polyubiquitin chains linked through the canonical Lys48 and less common Lys29 linkages [28]. Previously, we infected these mice with the 22L prion strain and observed evidence of UPS impairment in brain regions with the greatest PrP^Sc^ deposition at end-stage disease [24]. UPS dysfunction is associated with multifactorial toxicity including aberrant mitochondrial quality control [3, 39], loss of synaptic plasticity [1, 19], amino acid shortage [38] and ER stress [18, 26, 35]. We therefore hypothesised that UPS impairment may occur early in the course of prion disease pathogenesis and thus contribute to a progressive loss of neuronal function. The aim of the present study was to determine the temporal onset of UPS dysfunction in RML prion-infected mice by quantifying Ub^G76V^-GFP reporter levels in different cell types over the time course of disease progression.

## Materials and methods

### Animals

Ub^G76V^-GFP/1 mice [28] are congenic on a C57BL/6N background and were maintained as heterozygotes by back-crossing to the same wild-type strain. Animals were housed in individually ventilated cages with dust-free autoclaved wood bedding and had free access to food and water. The biological services facility was maintained at a constant temperature of 19–23 °C with 55 ± 10 % humidity in a 12-h light/dark cycle. Following isolation of genomic DNA from an ear-punch, animals were genotyped by PCR at 4 weeks of age. Primers for Ub^G76V^-GFP/1 transgene: 5′-CCTACAGCTCCTGGGCAACGT-3′ and 5′-TCGACCAAGCTTCCCCACCAC-3′.

### Prion inoculation of mice

RML prion inoculum (I6200) was prepared and titrated as described previously [4]. Female mice were inoculated intra-cerebrally with 30 μl of 1 % RML prion-infected brain homogenate or 1 % uninfected brain homogenate (control) between 8 and 10 weeks of age. Groups of five mice were killed by exposure to rising concentration of CO_2_ at designated serial time points (8, 12 and 18 weeks post-inoculation) or at onset of end-stage disease (mean onset: 23 weeks post-inoculation). Brains were removed and divided sagitally with one hemisphere fixed in 10 % (v/v) buffered formal-saline (BFS) for 48 h at room temperature and the other hemisphere fixed in 4 % paraformaldehyde in Sorensen phosphate buffer overnight at 4 °C. A replicate cohort of animals were inoculated and culled in parallel to provide unfixed tissue for western blotting and proteasome activity assays.

### Burrowing behaviour testing

Burrows consisted of 200 mm long, 68 mm diameter black plastic tubes, raised at one end by a 30 mm plastic support. The lower end of the tube was sealed with a plastic cap. Mice were habituated by placing burrows filled with food pellets into group housed cages for two consecutive nights. For individual testing, burrows filled with 200 g food pellets were placed in single-housed cages 4 h before the dark cycle. The percentage of food pellets displaced over 24 h was calculated by measuring the weight of pellets left in the tube.

### Tissue preparation

BFS-fixed hemispheres were dehydrated by passing through a series of industrial methylated spirits (70, 90 and 100 %) and cleared in xylene. Brain tissue was impregnated with molten paraffin wax and embedded in the sagittal orientation. 4-µm tissue sections were prepared using a Leica RM2135 microtome, floated in water at 40 °C and mounted on Superfrost™ microscope slides (Thermo Scientific). Mounted sections were left to air dry at 37 °C for 2 h and 60 °C for 16 h.

PFA-fixed hemispheres were immersed in 30 % (w/v) sucrose for 72 h for cryoprotection and then in Optimal Cutting Temperature (OCT, Tissue Tek). 30 µm sagittal cryosections were prepared using a Bright OTF5000 cryostat and placed in a solution containing 30 % glycerol, 30 % ethylene glycol, 30 % ddH_2_O and 10 % phosphate buffer at pH 7.2.

### PrP^Sc^ immunohistochemistry

Paraffin-embedded tissue sections were pre-treated with Tris-Citrate EDTA buffer for antigen retrieval [42]. PrP^Sc^ deposition was visualised using anti-PrP monoclonal antibody ICSM35 (D-Gen Ltd; 1 in 1000) on automated immunohistochemistry staining machines (Ventana Benchmark, Roche, Burgess Hill, UK) using proprietary reagents, as previously described [42].

### Immunofluorescence staining of cryosections

Immunofluorescence staining was performed by pre-treating free-floating sections with 0.1 % Triton X-100 for 15 min and 1 M glycine for 30 min at room temperature. Sections were then incubated in blocking solution (1 % bovine serum albumin and 0.1 % Triton X-100) for 1 h. Next, sections were incubated with primary antibodies in blocking solution for 16 h at 4 °C (Table S1). Sections were then washed in PBS and incubated with secondary antibodies diluted in PBS for 1 h in the dark at room temperature (Table S2).

### RNA in situ hybridisation

4 micrometre paraffin-embedded sections were used to perform RNA in situ hybridisation using the QuantiGene viewRNA kit (Affymetrix). ViewRNA probe sets specific for *eGFP*, *Actb* and *Gapdh* sequences were designed by Affymetrix and RNA in situ hybridisation was performed according to manufacturer’s instructions.

### Image acquisition and analysis

Confocal images of RNA in situ hybridisation and immunofluorescent staining were acquired with a Zeiss LSM 710 confocal microscope equipped with 405, 458, 488, 514, 561 and 633 nm laser lines. For immunofluorescent staining experiments, three fields of view were imaged across the thalamic region using a Plan-Apochromat 20× objective. Six serial sagittal sections were imaged per animal, separated by 300 μm intervals. For RNA in situ hybridisation experiments, Fast Red (514 nm laser) and Fast Blue (633 nm laser) substrates were visualised in six fields of view across the thalamic region using a Plan-Apochromat 40×/1.4 Oil DIC objective. Zeiss Immersol™ 518F was used as imaging medium. Two consecutive sagittal sections were imaged per animal. All images were taken with constant gain and pinhole settings at a resolution of 1024 × 1024 pixels. Bright-field images of DAB-labelled and H & E-stained sections were acquired using the Leica SCN400F Slide Scanner at 40× magnification.

Confocal images were processed using Volocity^®^ software (PerkinElmer, version 6.1.1). To identify cells with accumulation of the Ub^G76V^-GFP reporter, anti-GFP staining intensity was measured in populations of NeuN- and GFAP-labelled objects. An intensity value of three standard deviations greater than the population mean for uninfected control mice at 8 weeks post-inoculation was set as the threshold for classifying a cell as Ub^G76V^-GFP-positive.

### CAD5 cell culture and methods

CAD5 cells were grown in OptiMEM, 10 % foetal bovine serum (FBS) and 1x penicillin/streptomycin. CAD5 cells were chronically infected with RML prions (ScCAD5) as described [14]. CAD5 and ScCAD5 cells were treated with the proteasome inhibitor lactacystin (1 μM), the proteasome activator IU1 (50 μM), or both lactacystin (1 μM) and IU1 (50 μM), in 0.5 % DMSO vehicle for 16 h. Vehicle-only control cultures were treated with 0.5 % DMSO for 16 h. Cells were collected in PBS and centrifuged at 10,000×*g* for 1 min.

### Proteasome activity assays

#### Ub^G76V^-GFP mouse brain tissue

Animals were culled by exposure to rising concentration of CO_2_. Brains were removed and the thalamus dissected in ice-cold PBS using a dissection microscope. 10 % (w/v) homogenates were prepared in ice-cold proteasome assay lysis buffer (50 mM Tris–HCl, 5 mM MgCl_2_, 250 mM sucrose, 2 mM ATP at pH 7.4) and centrifuged at 13,000×*g* for 20 min at 4 °C. Resulting supernatants were placed on ice and total protein concentrations measured using the Bio-Rad Protein Assay. For determination of proteasome activity, supernatants were adjusted to 1 mg/ml total protein by dilution in proteasome assay lysis buffer supplemented with 1 mM DTT. To measure the rate of hydrolysis, 10 μg sample was incubated with 100 μM of a fluorogenic peptide substrate in 100 μl proteasome assay reaction buffer (50 mM Tris–HCl, 5 mM MgCl2, 1 mM DTT, 2 mM ATP at pH 7.4). Chymotrypsin-like activity was determined using the substrate Suc-LLVY-aminomethylcoumarin (AMC) (Enzo Life Sciences); caspase-like activity was determined using the substrate Ac-Nle-Pro-Nle-Asp-AMC (Bachem) and trypsin-like activity was determined using the substrate Boc-Leu-Arg–Arg-AMC (Enzo Life Sciences). Samples were incubated for 1 h at 37 °C and the release of AMC measured at 1-min intervals in a TECAN 96-well plate reader (360 nm excitation; 465 nm emission). All assays were performed in triplicate. Background activity caused by non-proteasomal degradation was determined by addition of 5 μM epoxomicin (Enzo) for 30 min at 37 °C. Mean fluorescence values were calculated per sample by measuring the mean fluorescence generated in the linear reaction phase across triplicate wells. To calculate specific proteasome catalytic activities, mean fluorescence values of epoxomicin-treated controls were subtracted from each sample. Catalytic activity was then expressed as a percentage, relative to the mean of the control-inoculated group.

#### CAD5 cells

CAD5 or ScCAD5 cells were treated with IU1 (50 μM) or 0.5 % DMSO vehicle for 16 h, collected in ice-cold PBS and centrifuged at 10,000×*g* for 1 min. Cells were re-suspended in proteasome assay lysis buffer and passed through a 27G gauge needle ten times. The homogenate was then centrifuged at 20,000×*g* for 20 min. The proteasome activity of the resulting supernatants was determined as described above. IU1 (50 μM) was included in the reaction as appropriate.

### Western blot analysis

#### Mouse brain tissue

Animals were culled by exposure to rising concentration of CO_2_. Brains were removed and the thalamus dissected in ice-cold PBS using a dissection microscope. 10 % (w/v) homogenates were prepared in ice-cold tissue lysis buffer (100 mM Tris–HCl, 100 mM NaCl, 1 % Triton X-100, 10 mM EDTA at pH 7.4) supplemented with cOmplete Mini protease and PhosSTOP phosphatase inhibitors (Roche) and centrifuged at 13,000×*g* for 20 min at 4 °C. The resulting supernatants were stored on ice and total protein concentrations determined using the Bio-Rad Protein Assay. Samples were adjusted to 1 mg/ml total protein concentration with lysis buffer and 3× SDS-PAGE loading buffer (BioVision), and incubated at 96 °C for 5 min. Lysates (20 μg) were separated on 4–20 % SDS gels and probed with antibodies to PSMA5, PSMD1, polyubiquitinated proteins (clone FK1; Millipore) and β-actin (Sigma). Immunoblots were developed using IRDye secondary antibodies and the LI-COR Odyssey scanner.

#### CAD5 cells

Cell pellets were re-suspended in ice-cold lysis buffer (1 % Triton X-100, 0.5 % sodium deoxycholate in PBS supplemented with benzonase (500 units per ml) and prepared for SDS-PAGE as described previously [12]. Immunoblots were probed with the following antibodies: anti-PrP (ICSM35, 1:8000), anti-polyubiquitin (clone FK1, 1:1000; Millipore) and anti-ERK 1 and 2 (1:5000; Cell Signalling). Immunoblots were developed using IRDye secondary antibodies and the LI-COR Odyssey scanner.

### Dot blot analysis

Brain tissue was prepared as described for Western blot analysis, with subsequent measurement of total protein concentrations using the Bio-Rad Protein Assay. Samples were adjusted to 1 mg/ml total protein concentration with lysis buffer and 3× SDS-PAGE loading buffer (BioVision), and incubated at 96 °C for 5 min. Lysates (20 μg) were applied to a nitrocellulose membrane using Bio-Dot Microfiltration Apparatus (Bio-Rad), following the manufacturer’s instructions. The membranes were probed with an anti-ubiquitin antibody (clone FK1; Millipore) and analysed using the LI-COR Odyssey Infra-Red Imaging System, following the manufacturer’s instructions.

### Data analysis

In all in vivo experiments, at least three animals per group were analysed and data presented as percentage mean ± SEM. All data analysis was performed using GraphPad Prism software (GraphPad Software Inc.; version 6). Burrowing behaviour was analysed by comparing the percentage food pellets displaced by control and prion-infected mice at serial time points using a mixed model ANOVA with Bonferroni post hoc tests. The percentages of Ub^G76V^-GFP-positive cells across serial time points in control- and prion-infected groups were compared using a two-way ANOVA with Holm–Sidak-corrected post hoc *t* tests. In RNA in situ hybridisation experiments, relative Ub^G76V^-GFP mRNA levels were calculated by normalising the mean Ub^G76V^-GFP staining intensity to the mean staining intensity of the *Actb* or *Gapdh* control transcripts, and subsequently comparing control- and prion-infected groups using a two-tailed Student’s *t* test. In proteasome activity assays, fluorescence levels were calculated by measuring the mean fluorescence generated in the linear reaction phase across triplicate wells. To calculate specific proteasome catalytic activities, mean fluorescence values of epoxomicin-treated controls were subtracted from each sample and subsequently expressed as a percentage of the control group mean. Proteasome activity was compared between treatment groups using a two-tailed Student’s *t* test (Fig. 7a) or one-way ANOVA with Bonferroni post hoc tests (Fig. 7b). For proteasome subunit quantification experiments, LI-COR scanned immunoblots were converted to grey-scale images and signal intensity compared by two-tailed Student’s *t* tests. To compare total ubiquitin levels in brain extracts of prion- and control-infected mice across the disease course, LI-COR scanned dot blots were converted to grey-scale images and signal intensities compared using a two-way ANOVA with Holm–Sidak-corrected post hoc *t* tests.

## Results

### Progression of behavioural deficits and neuropathology in prion-infected Ub^G76V^-GFP mice

**Fig. 1:**
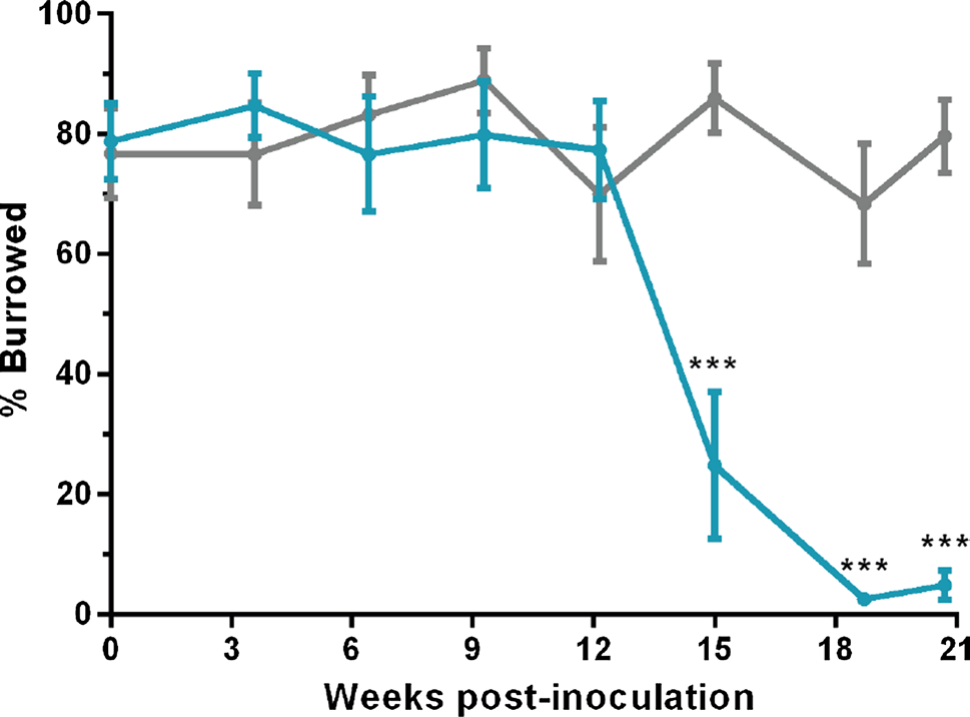
Prion-infected Ub^G76V^-GFP reporter mice display sudden decline in burrowing behaviour from 15 wpi. Ub^G76V^-GFP reporter mice were inoculated with 30 μl of 1 % RML prion-infected brain homogenate or 1 % uninfected brain homogenate at 8–10 weeks of age. Burrowing behaviour was assessed by measuring the percentage of food pellets displaced from a plastic burrow placed in single-housed cages overnight. Significant impairment of burrowing behaviour was observed from 15 wpi in prion-infected animals (*blue line*) versus uninfected controls (*grey line*). Data are percentage mean ± SEM (***p < 0.001; mixed model ANOVA with Bonferroni post hoc tests; *n* = 9 per group)

To understand the pattern of neurodegeneration in prion-infected Ub^G76V^-GFP mice, groups of female mice were inoculated with RML prions, or uninfected control homogenate, at 8–10 weeks of age. Burrowing behaviour, a highly sensitive measure of the early effects of prion infection [6], was assessed every 3 weeks by measuring the proportion of food pellets displaced from a plastic tube overnight. The earliest evidence of burrowing impairment was observed at 15 weeks post-inoculation (wpi), when prion-infected Ub^G76V^-GFP mice displaced 60 % fewer food pellets than control animals (Fig. 1). A complete loss of burrowing behaviour was observed at 18 wpi, demonstrating the rapid nature of prion-mediated neurodegeneration. In accordance with animal welfare regulations, mice were killed at the onset of ataxia, sustained hunched posture, significant alteration in breathing rate or loss of the righting reflex. The mean disease incubation time in prion-infected Ub^G76V^-GFP mice was 23 wpi, with no significant difference compared to wild-type littermate controls (Fig. S1), confirming that disease progression was independent of the Ub^G76V^-GFP transgene.

**Fig. 2:**
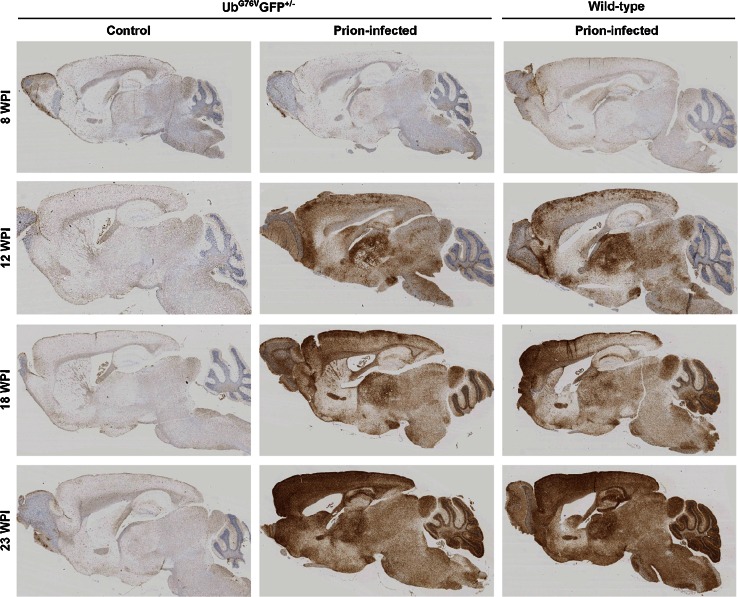
PrP^Sc^ distribution in brains of prion-infected Ub^G76V^-GFP mice and wild-type littermates. Ub^G76V^-GFP mice were inoculated with 30 μl of 1 % RML prion-infected brain homogenate or 1 % uninfected brain homogenate (control) between 8 and 10 weeks of age. Animals were culled at 8, 12, 18 and 23 wpi. To control for the presence of the Ub^G76V^-GFP transgene, an additional group of wild-type littermates received RML prion inocula. Staining of formic acid treated sections reveals intense PrP^Sc^ immunostaining from 12 wpi with widespread distribution in the thalamus and focal regions of the cortex. A similar spatiotemporal pattern of PrP^Sc^ accumulation was observed in all prion-infected animals, independent of genotype. Data are representative of *n* = 4 mice per group

To study disease progression by neuropathology, groups of age-matched prion-infected and control mice were also culled at 8, 12 and 18 wpi. Brains were examined histologically to determine the course of neuropathological changes in prion-infected Ub^G76V^-GFP mice. By 12 wpi, initial PrP^Sc^ deposition was observed throughout the thalamus and in selected regions of the cortex and hypothalamus of prion-infected mice (Fig. 2). Less intense, diffuse immunostaining was visualised in regions of the midbrain and brainstem. By 18 wpi, PrP^Sc^ accumulation became more pronounced in these regions, with evidence of spread to the cerebellum. By end-stage disease at 23 wpi, dense PrP^Sc^ deposition was detectable in all brain regions examined. No differences in the spatiotemporal pattern of PrP^Sc^ distribution were observed between prion-inoculated Ub^G76V^-GFP mice and their wild-type littermates. As reported previously, the thalamus displayed robust PrP^Sc^ accumulation at early stages of disease [33]. As a result, this brain region was selected for phenotype analysis in subsequent experiments.

**Fig. 3:**
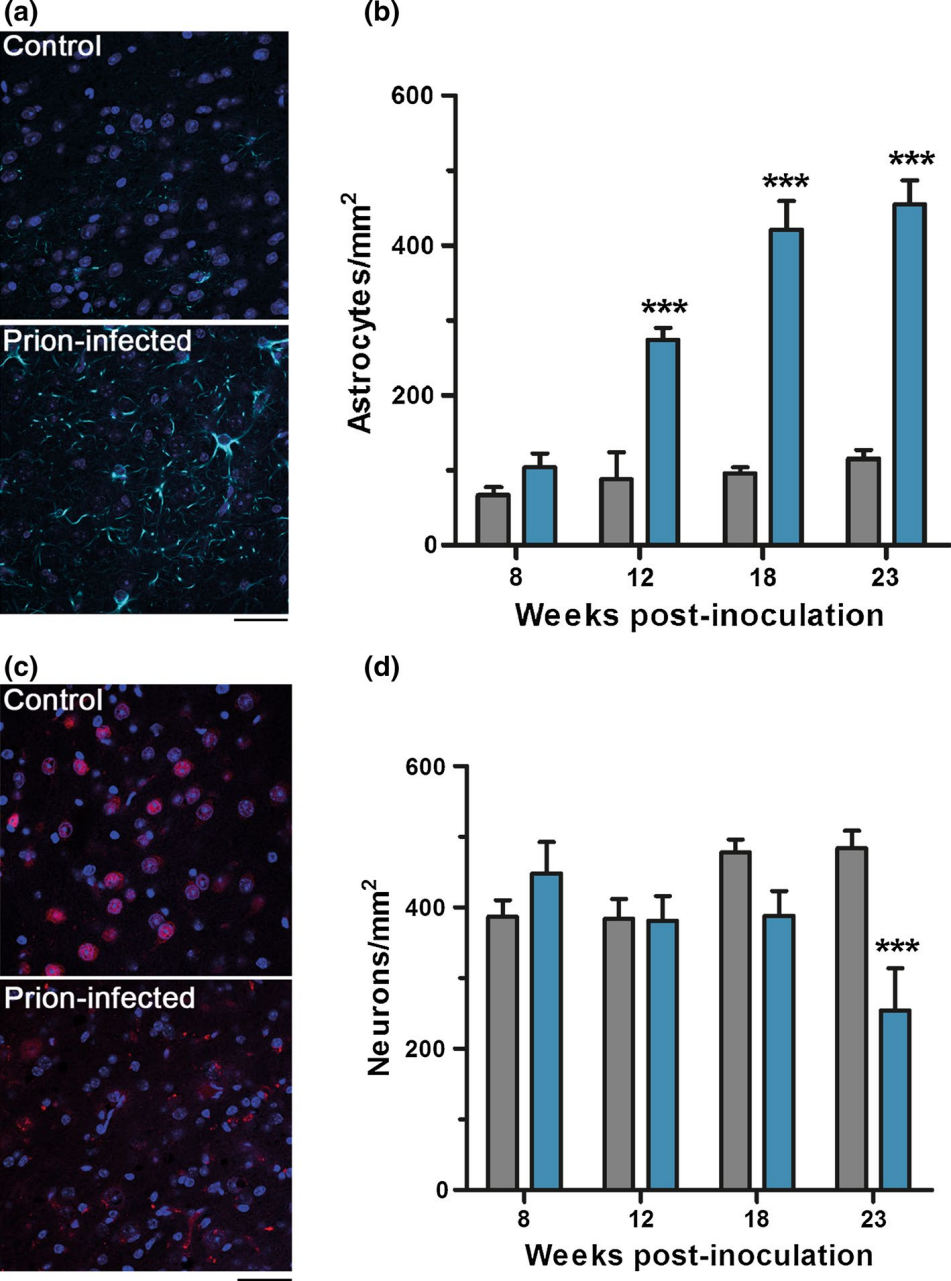
Reactive astrocytosis and neuronal loss in the thalamus of prion-infected Ub^G76V^-GFP reporter mice. Ub^G76V^-GFP reporter mice were inoculated with 30 μl of 1 % RML prion-infected brain homogenate or 1 % uninfected brain homogenate (control) between 8 and 10 weeks of age (*n* = 3–4 per group). Animals were culled at 8, 12, 18 and 23 wpi. **a** Anti-GFAP immunofluorescent staining (*cyan*) of frozen sections reveals reactive astrocytosis at 12 wpi in the thalamus. **b** Quantification of GFAP-labelled cells in the thalamus of control (*grey bars*) and prion-infected (*blue bars*) Ub^G76V^-GFP mice. Extensive proliferation of reactive astrocytes is observed from 12 wpi. **c** Anti-NeuN immunofluorescent staining (*red*) of frozen sections reveals marked neuronal loss at 23 wpi. **d** Quantification of NeuN-labelled cells in the thalamus of control (*grey bars*) and prion-infected (*blue bars*) Ub^G76V^-GFP mice. A significant reduction in neuronal density is observed at 23 wpi. Data are mean ± SEM (***p < 0.001; two-way ANOVA with Holm Sidak-corrected post hoc *t* tests).* Scale bar* in **a**, **c** = 40 μm. Nuclei were stained with DAPI (*blue*)

Regional deposition of PrP^Sc^ is known to be a strong inducer of reactive astrocytosis, characterised by the accumulation of GFAP, cytoplasmic hypertrophy and the protrusion of long processes into the surrounding interstitium [11]. Quantification of GFAP-labelled cells in the thalamus revealed progressive reactive astrocytosis in prion-infected mice from 12 wpi (Fig. 3a, b). Spongiform vacuolation of the neuropil, a classical histological feature of prion disease, was observed in the thalamus from 18 wpi (Fig. S2). Histological analysis of NeuN-labelled cells revealed a 50 % reduction in neuronal density in the thalamus at end-stage disease, coincident with the onset of terminal motor signs (Fig. 3c, d).

### Ub^G76V^-GFP reporter accumulates early in prion-infected mice

**Fig. 4:**
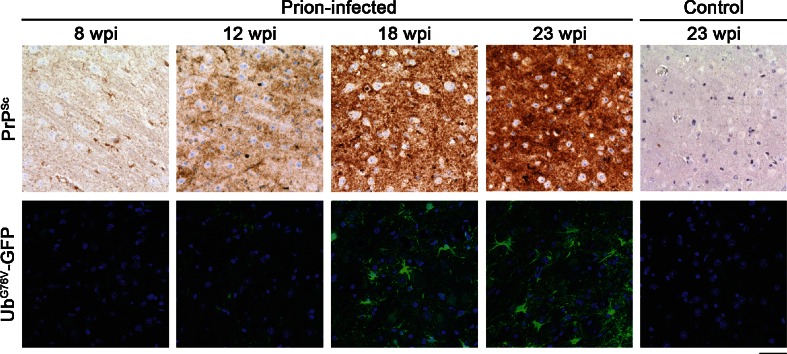
Early accumulation of the Ub^G76V^-GFP reporter in prion-infected mice. Ub^G76V^-GFP reporter mice were inoculated with 30 μl of 1 % RML prion-infected brain homogenate or 1 % uninfected brain homogenate (control) between 8 and 10 weeks of age (*n* = 3–4 per group). Animals were culled at 8, 12, 18 and 23 wpi. Prion protein staining of formic acid treated sections reveals PrP^Sc^ immunostaining from 12 wpi in thalamus. Immunofluorescent staining of frozen sections with anti-GFP antibody reveals progressive accumulation of Ub^G76V^-GFP reporter from 12 wpi. Ub^G76V^-GFP reporter levels remained unchanged in age-matched uninfected control mice. *Scale bar* 40 μm

**Fig. 5:**
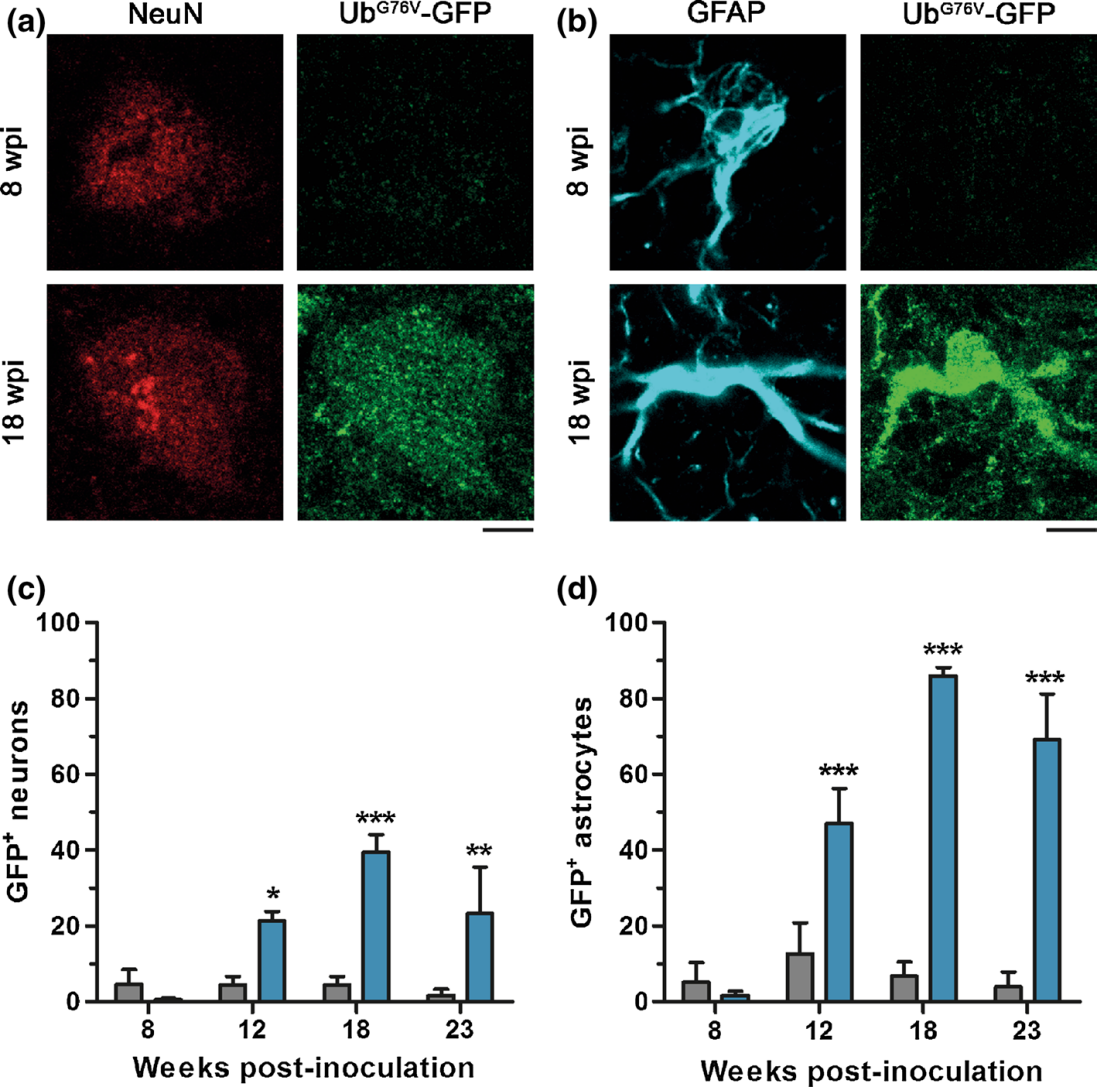
Ub^G76V^-GFP reporter accumulates in neurons and astrocytes of prion-infected mice. Ub^G76V^-GFP reporter mice were inoculated with 30 μl of 1 % RML prion-infected brain homogenate or 1 % uninfected brain homogenate (control) between 8 and 10 weeks of age (*n* = 3–4 per group). Animals were culled at 8, 12, 18 and 23 wpi. **a** Comparison of immunofluorescent staining with anti-GFP and anti-NeuN antibodies in thalamus of prion-infected Ub^G76V^-GFP reporter mice at 8 wpi versus 18 wpi. *Scale bar* 3.75 μm. **b** Comparison of immunofluorescent staining with anti-GFP and anti-GFAP antibodies in thalamus of prion-infected Ub^G76V^-GFP reporter mice at 8 wpi versus 18 wpi. *Scale bar* 6.5 μm. **c** Quantification of GFP levels in NeuN-labelled cells of uninfected (*grey bars*) and prion-infected (*blue bars*) Ub^G76V^-GFP mice. Ub^G76V^-GFP reporter accumulates in a subset of neurons from 12 wpi. **d** Quantification of GFP levels in GFAP-labelled cells of uninfected (*grey bars*) and prion-infected (*blue bars*) Ub^G76V^-GFP mice. Ub^G76V^-GFP reporter accumulates in astrocytes from 12 wpi. Data are percentage mean ± SEM (*p < 0.05; **p < 0.01;***p < 0.001; two-way ANOVA with Holm–Sidak-corrected post hoc *t* tests)

To determine the functional status of the UPS at each stage of disease progression, Ub^G76V^-GFP levels were assessed by anti-GFP immunofluorescent staining. Within the time window of the present study, no age-related impairment of the UPS was observed since numbers of GFP-positive cells remained persistently low in control-inoculated mice. A progressive increase in Ub^G76V^-GFP levels was observed in the thalamus of prion-infected mice from 12 wpi (Fig. 4). Ub^G76V^-GFP reporter accumulation was also observed in other brain regions affected by PrP^Sc^ deposition (Fig. S3); however, subsequent phenotype analysis focussed on the thalamus due to the robust PrP^Sc^ accumulation observed at early disease stages (Fig. 2). To identify which cell populations were affected by UPS dysfunction, we double-labelled cells with specific marker antibodies for the different neural cell populations. A significant increase in the percentage of GFP-positive neurons was observed in the thalamus of prion-infected mice at 12 wpi (Fig. 5a, c). This preceded the onset of burrowing behaviour deficits and coincided with the first appearance of astrocytosis. By 18 wpi, this proportion had increased, with 40 % of neurons displaying accumulation of the GFP reporter. By end-stage disease, a large amount of variation in the percentage of GFP-positive neurons was observed in prion-infected mice. This could indicate variation in the recognition of end-stage prion disease based on current phenotypic criteria or reflect the significant neuronal loss observed at this time point.

In addition to evidence of neuronal UPS dysfunction, marked accumulation of the GFP reporter was also observed in the reactive astrocytes of prion-infected mice (Fig. 5b, d). At 12 wpi, 47 % of GFAP-labelled astrocytes were identified as GFP-positive. This proportion increased dramatically to more than 86 % of reactive astrocytes by 18 wpi. Dual immunofluorescence staining of GFP and the microglial marker Iba1 revealed no evidence of GFP-reporter accumulation in microglia at any time point investigated.

Interestingly, a reduction in the proportion of GFP-positive neurons and astrocytes was observed at end-stage disease. While the reason for this trend remains unclear, one possible explanation is translational repression secondary to sustained activation of the unfolded protein response in prion-infected mice [34]. This could reduce the proportion of GFP-positive cells by reducing de novo synthesis of Ub^G76V^-GFP protein and by reducing the burden of improperly folded nascent proteins targeted to the proteasome via the ERAD pathway.

To demonstrate that the accumulation of the Ub^G76V^-GFP protein was a result of impaired protein degradation rather than increased protein synthesis, we measured *Ub*^*G76V*^-*GFP* transcript levels by RNA in situ hybridisation at 18 wpi, when accumulation of the Ub^G76V^-GFP protein is well established and anatomically widespread (Fig. S4). No differences in *Ub*^*G76V*^-*GFP* transcript levels were observed in the thalamus of control and prion-infected Ub^G76V^-GFP reporter mice at 18 wpi, confirming that the increase in Ub^G76V^-GFP protein levels in diseased animals is due to impaired degradation of the reporter rather than elevation/stabilisation of the Ub^G76V^-GFP transcript.

### Accumulation of polyubiquitinated conjugates in neurons of prion-infected mice

**Fig. 6:**
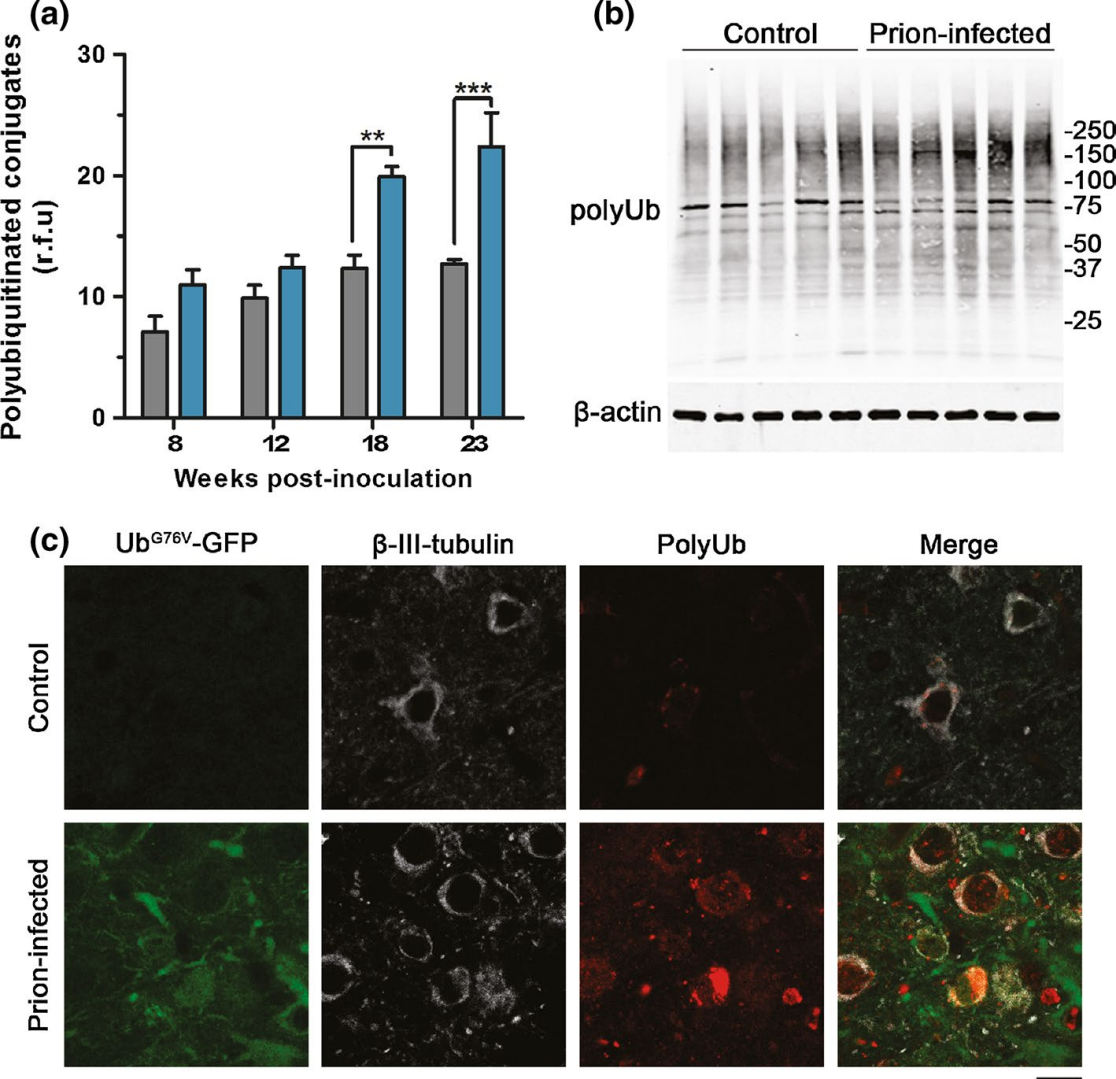
Polyubiquitinated conjugates accumulate in brains of prion-infected mice. **a** Dot blot quantification of polyubiquitinated conjugate levels in whole brain homogenates of prion-infected (*blue bars*) and control-inoculated (*grey bars*) wild-type mice. Original dot blot image shown in Fig. S5. Data are normalised to β-actin expression and expressed as mean ± SEM (*n* = 4 per group; **p < 0.01;***p < 0.001; two-way ANOVA with Holm–Sidak-corrected post hoc *t* tests). **b** Thalamic tissue from prion-infected and uninfected control Ub^G76V^-GFP mice at 23 wpi was homogenised and analysed by anti-polyubiquitin western blot (clone FK1). Densitometry revealed a 53 % increase in levels of high MW polyubiquitinated conjugates (>100 kDa) in prion-infected Ub^G76V^-GFP mice, compared with uninfected controls when normalised to the β-actin housekeeping gene (*p* < 0.01; two-tailed Student’s *t* test; *n* = 5 per group). **c** Frozen brain sections from prion-infected and uninfected control mice at 23 wpi were stained with anti-polyubiquitin (*red*), anti-GFP (*green*) and anti-β-III-tubulin (*white*) antibodies. Prion-infected mice show increased levels of Ub^G76V^-GFP reporter and polyubiquitin staining in β-III-tubulin-positive neurons. Scattered polyubiquitin-positive puncta are seen throughout the neuropil in prion-infected mice. *Scale bar* 10 μm

To investigate whether observed UPS dysfunction is associated with impaired substrate clearance, we conducted a dot blot analysis of polyubiquitinated conjugate levels in brains of prion-infected and uninfected control mice at different stages of disease progression (Fig. S5). This experiment was performed using brain tissue from wild-type mice, to allow comparison of endogenous UPS substrate levels, independently from the polyubiquitinated Ub^G76V^-GFP reporter. A significant increase in levels of polyubiquitinated conjugates was observed from 18 wpi in prion-infected animals (Fig. 6a). These findings were confirmed by anti-polyubiquitin western blot of thalamic tissue from control and prion-inoculated Ub^G76V^-GFP reporter mice (Fig. 6b). Densitometry revealed a 53 % increase in levels of high molecular weight (MW) polyubiquitinated conjugates in prion-infected Ub^G76V^-GFP reporter mice. Subsequent immunofluorescence staining of frozen brain sections revealed localised deposition of polyubiquitin in thalamic neurons of prion-infected Ub^G76V^-GFP reporter mice at end-stage disease (Fig. 6c). Taken together, these observations demonstrate that observed UPS impairment is associated with marked disruption of neuronal proteostasis in prion-infected Ub^G76V^-GFP reporter mice.

Interestingly, Ub^G76V^-GFP reporter accumulation was only observed in neurons with high levels of polyubiquitin staining (Fig. 6c). This suggests a possible discrepancy in the detection sensitivity of the Ub^G76V^-GFP reporter versus polyubiquitin. Alternatively, the accumulation of polyubiquitin in the absence of Ub^G76V^-GFP in some neurons may indicate prion-mediated disruption of non-proteolytic pathways which are also regulated by post-translational polyubiquitination.

### Impairment of proteasome catalytic activity in prion-infected mice

**Fig. 7:**
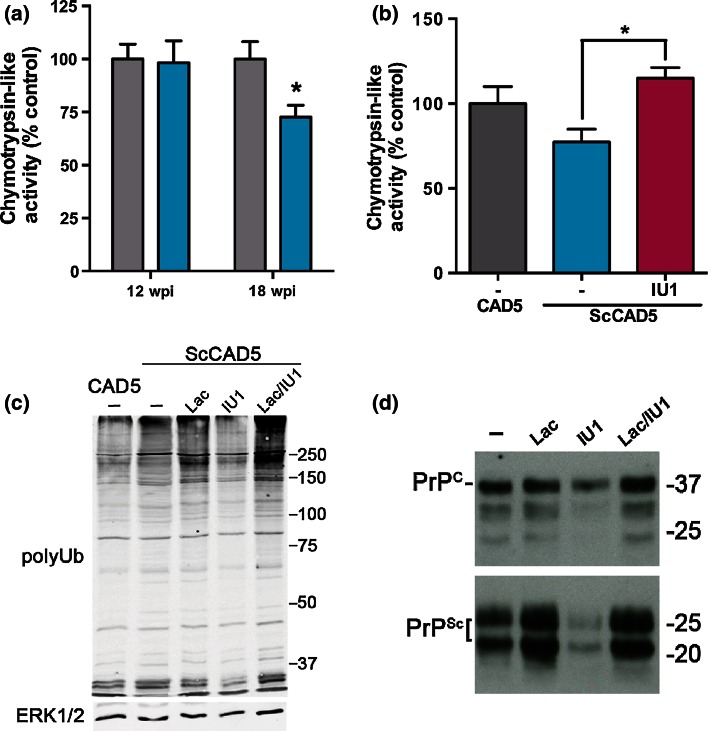
Measurement of 26S proteasome activity in prion-infected Ub^G76V^-GFP reporter mice and ScCAD5 cells. **a** Ub^G76V^-GFP reporter mice were inoculated with 30 μl of 1 % RML prion-infected brain homogenate or 1 % uninfected brain homogenate (control) and culled at 12 wpi or 18 wpi. Thalamic tissue was homogenised and chymotrypsin-like activity assessed by measuring fluorescence generated from cleavage of a site-specific peptide substrate, adjusted to an epoxomicin-treated control. Data are percentage mean ± SEM, expressed relative to control (*n* = 5 per group). A significant impairment in chymotrypsin-like activity was observed in prion-infected mice at 18 wpi (*p < 0.05; two-tailed Student’s *t* test). No significant difference in caspase- or trypsin-like activities was observed (Fig. S6**)**. **b**–**d** Prion-infected (ScCAD5) and uninfected control (CAD5) cells were treated with either 1 μM lactacystin (Lac), 50 μM IU1 (IU1), 1 μM lactacystin and 50 μM IU1 (Lac/IU1), or DMSO vehicle alone (-) for 16 h. An equal concentration of 0.5 % DMSO vehicle was used in all cultures. **b** Cell lysates were collected and chymotrypsin-like proteasome activity analysed by fluorogenic assay. Data are percentage mean ± SEM, expressed relative to CAD5 vehicle-only (-) control (*n* = 4 per group). Prion infection results in a modest decrease in proteasome activity, which is reversed by IU1 (*p < 0.05; one-way ANOVA with Bonferroni post hoc tests). **c** Cell lysates were analysed by immunoblotting with anti-polyubiquitin antibody (FK1). ERK1/2 was used as a loading control. Changes in polyubiquitin levels are closely related to underlying proteasome activity. Combined lactacystin and IU1 treatment increased polyubiquitinated protein content, consistent with the inhibition of de-ubiquitinase USP14 by IU1. **d** Cell lysates were collected and equal quantities analysed by immunoblotting with anti-PrP antibodies (untreated or following limited proteinase K digestion to reveal PrP^Sc^). Inhibition of the proteasome results in accumulation of PrP^Sc^; stimulation of proteasome activity reduces PrP^Sc^ load in ScCAD5 cells

To examine whether observed UPS dysfunction could be attributed to impairment of the proteasome itself, we compared proteasome catalytic activities in control and prion-inoculated Ub^G76V^-GFP reporter mice at early and late stages of disease progression. Catalytic turnover by the three peptidase sites of the 20S proteasome was measured by comparing the rates of hydrolysis of specific fluorogenic peptides in homogenised thalamic tissue. At 12 wpi, no significant differences in chymotrypsin-, caspase- or trypsin-like activities were observed (Fig. 7a, Fig. S6). Since only 21 % of thalamic neurons displayed Ub^G76V^-GFP reporter accumulation at this early disease stage (Fig. 5c), it is likely that measurement of total proteasome activity in a crude thalamic homogenate lacked the sensitivity to detect catalytic impairment in the small subpopulation of affected cells. We therefore also measured proteasome catalytic activities at 18 wpi, when Ub^G76V^-GFP reporter accumulation was more anatomically widespread throughout the thalamus (Fig. 4). At this stage, we observed a 27 % reduction in the activity of the chymotrypsin-like site in the thalamus of prion-infected mice (Fig. 7a). No evidence of impairment was observed for caspase- or trypsin-like sites in the disease condition (Fig. S6). Impairments in proteasome peptidase activity could be masked by a compensatory increase in the total number of proteasomes. To confirm that the size of the proteasome pool remained unaltered in prion-infected mice, levels of two key constitutive proteasome subunits were measured by western blot. At 18 wpi, levels of PSMD1, a subunit of the 19S regulatory particle and PSMA5, a subunit of the 20S proteasome, were unaltered in the thalamus of prion-infected mice (Fig. S7). Collectively, these results suggest that there is selective inhibition of the chymotrypsin-like site of the proteasome in prion-infected mice, independent of any overall changes in proteasome number.

### Proteasome activator IU1 reduces levels of polyubiquitinated conjugates and PrP^Sc^ in prion-infected cells

To test whether up-regulation of proteasome activity could facilitate clearance of the backlog of polyubiquitinated conjugates observed in the present study, we tested the effect of the small molecule Usp14 inhibitor IU1 in prion-infected CAD5 neuronal cells. Infection of CAD5 cells with RML prions resulted in a modest decrease in proteasome activity (Fig. 7b), consistent with previously described observations in PK1 cells and prion-infected Ub^G76V^-GFP mice [24]. Treatment of prion-infected CAD5 cells with the proteasome inhibitor lactacystin resulted in increased levels of polyubiquitinated conjugates (Fig. 7c) and further accumulation of PrP^Sc^ (Fig. 7d). This could be attributed to a small rise in levels of PrP^C^ substrate or impaired clearance of misfolded PrP conformers in lactacystin-treated cells. In contrast, activation of the proteasome by treatment with the Usp14 inhibitor IU1 (Fig. 7b), decreased levels of polyubiquitinated conjugates (Fig. 7c) and resulted in a marked reduction in PrP^Sc^ load (Fig. 7d). Importantly, these effects were reversed by the inclusion of lactacystin, verifying that the action of IU1 is dependent upon proteasome activity. As expected, combined lactacystin and IU1 treatment increased total polyubiquitinated protein content, consistent with the inhibition of de-ubiquitinase Usp14 by IU1. Taken together, these findings suggest that the proteasome plays an important role in the clearance of polyubiquitinated conjugates and regulation of PrP^Sc^ levels in prion-infected neuronal cells.

## Discussion

In the present study, we identify functional impairment of the UPS as an early feature of prion disease pathogenesis, preceding the onset of behavioural dysfunction and neuronal loss. Progressive UPS impairment in neuronal and astrocytic cell populations was accompanied by marked accumulation of polyubiquitinated conjugates. Consistent with a role of UPS dysfunction in prion disease pathogenesis, pharmacological activation of the UPS in prion-infected cultured cells facilitated clearance of polyubiquitinated conjugates and reduced total levels of PrP^Sc^. Taken together, these findings identify the UPS as an important target in the development of therapeutics for the treatment of prion diseases.

Impairment of the UPS is likely to have pleiotropic effects in the prion-infected brain, which together could contribute to progressive neurotoxicity, synaptic dysfunction and ultimately cell death [31]. Previous studies of murine scrapie reported that early impairment of burrowing behaviour is coincident with a marked decline in levels of pre- and post-synaptic proteins [5, 16, 34]. Mallucci and colleagues identified that this synaptic failure correlated with sustained translational repression of global protein synthesis by eIF2α-P, a key mediator of the unfolded protein response (UPR; Moreno et al. [34]). Precipitant ER stress was attributed to rising levels of misfolded PrP in the ER. In the present study, neuronal UPS impairment preceded the earliest behavioural signs of synaptic dysfunction, suggesting that UPR activation may also arise from reduced clearance of ubiquitinated substrates by the ER-associated protein degradation (ERAD) pathway and associated accumulation of non-native proteins in the ER.

In addition to disruption of cellular proteostasis, UPS dysfunction may lead to neurotoxicity through activation of pro-apoptotic pathways. In cellular models of prion disease, intra-neuronal prion propagation in the presence of mild proteasome inhibition triggered formation of cytosolic aggresomes containing vimentin, PrP^Sc^, hsp70, ubiquitin and proteasome subunits [8, 25]. The PrP^Sc^ aggresome formation was temporally associated with caspase 3 and 8 activation, triggering neuronal apoptosis [25]. Interestingly, abrogation of aggresome formation with microtubule inhibitors prevented apoptosis, suggesting that the sequestration of key components of the cellular protein quality control machinery may induce neurotoxicity through depletion of functional soluble pools and/or the formation of insoluble protein aggregates. Co-precipitation of vimentin and PrP^Sc^ in the brains of prion-infected mice suggest that similar aggresome-like structures may also be present in vivo [25]. Together with evidence of early neuronal UPS dysfunction described in the present study, these observations indicate that impairment of the UPS may promote neuronal apoptosis in regions worst affected by prion pathology.

Despite early occurrence of UPS dysfunction in thalamic neurons in the present study, significant neuronal loss was not observed until end-stage disease (Fig. 3c). This apparent discrepancy could be accounted for by the compensatory induction of other protein quality control systems. For example, the accumulation of autophagic vacuoles has been described in neurons of scrapie-infected hamsters and in synapses of human CJD and FFI patients [2, 36]. More recently, Xu and colleagues reported that late-stage activation of the macroautophagic system in scrapie-infected hamsters was associated with a reduction in the levels of polyubiquitinated protein conjugates and the macroautophagy substrate sequestosome 1 [44]. Compensatory up-regulation of macroautophagy may help to reduce proteotoxic stress in neurons with UPS dysfunction, resulting in a more protracted disease course and delayed onset of neuronal loss.

In addition to the neuronal cell population, we observed marked accumulation of the Ub^G76V^-GFP reporter in surrounding reactive astrocytes. Despite apparent disruption to cellular proteostasis, extensive astrocyte proliferation was observed across the disease course, with no evidence of a reduction in astrocyte counts at end-stage disease. The relative resistance of astrocytes to the cytotoxic effects of proteasome impairment has been reported by numerous studies [10, 21, 32, 40, 46], indicating that astrocytes may have well adapted protein quality control pathways to compensate for UPS failure. A recent study of scrapie infection in hamsters reported a selective increase in αβ-crystallin expression in astrocytes [43]. Similar observations were reported in regions of severe pathology in human CJD and FFI post-mortem brain tissue [43]. Thus, the efficient induction of heat shock response genes in astrocytes may account for their apparent resistance to high levels of UPS dysfunction in the present model.

The aetiology of UPS dysfunction in the brains of prion-infected mice could partly be attributed to a reduction in activity of the 26S proteasome. Inhibition of chymotrypsin- and caspase-like activities was previously identified in the brains of RML and ME7 prion-infected mice at end-stage disease [22, 24]. Subsequent in vitro studies suggested that misfolded PrP isoforms inhibit proteolytic activity by stabilising the closed conformation of the substrate entry channel [7]. To interact with the proteasome, a cytosolic form of PrP^Sc^ must be present in brains of prion-infected mice. The existence of such a species is supported by evidence of PrP^Sc^ and proteasome colocalisation in perinuclear aggresomes in prion-infected neuroblastoma cells [25] and co-immunoprecipitation of PrP^Sc^ with vimentin [25] and subunits of the 26S proteasome [7] in prion-infected mouse brains. The trafficking pathway by which Pr^Sc^ gains access to the cytosolic compartment remains the focus of ongoing research (reviewed in [13]).

In the present study, we measured proteasome catalytic activities in the thalamus 5 weeks before end-stage disease, when a maximal proportion of neurons and astrocytes displayed Ub^G76V^-GFP reporter accumulation (Fig. 5). Selective impairment of chymotrypsin-like activity was observed, in the absence of any detectable change in the caspase- or trypsin-like sites. Whilst previous in vitro studies have suggested that simultaneous inhibition of multiple catalytic activities is required to disrupt proteasome function [23], it remains unclear whether impairment of a single activity is sufficient to have detrimental effect in the context of protein-misfolding disorders. In addition to impairment of 26S proteasome catalytic activity, UPS dysfunction observed in the present study could be attributed to proteasomal insufficiency [29], whereby ongoing prion propagation could overwhelm the capacity of the proteasome to clear misfolded protein substrates arising from errors in translation or co-translational folding.

To establish whether enhancement of proteasome activity could promote clearance of the backlog of polyubiquitinated conjugates, we tested the effect of the small molecule inhibitor IU1 in prion-infected CAD5 (ScCAD5) neuronal cells. Upon RML prion infection, these cells display impairment of chymotrypsin-like proteasome activity and accumulation of polyubiquitinated conjugates, mirroring observations in prion-infected Ub^G76V^-GFP reporter mice. IU1 has previously been shown to enhance proteasome activity by inhibiting ubiquitin chain trimming mediated by the proteasome-associated deubiquitinating enzyme Usp14 [27]. Suppression of chain trimming is thought to stabilise ubiquitinated substrates on the proteasome and promote their unfolding and translocation into the 20S core particle for degradation. We observed that treatment of ScCAD5 cells with IU1 led to a reduction in levels of polyubiquitinated conjugates, suggesting that UPS up-regulation is sufficient to reduce levels of potentially toxic ubiquitinated proteins which accumulate in prion infection.

Furthermore, stimulation of UPS activity was sufficient to reduce cellular levels of PrP^Sc^. Consistent with previous reports in prion-infected N2a cells [17], we observed that IU1 treatment resulted in a significant reduction in PrP^C^ levels in ScCAD5 cells (Fig. 7d). Whilst the major route of PrP^C^ degradation is considered to be the endosomal/lysosomal pathway [15], a subpopulation of PrP^C^ is also known to be degraded by ER-associated degradation (ERAD), prior to its release into the secretory pathway [30, 45]. Activation of the proteasome as the final step of the ERAD pathway could therefore account for reduced PrP^C^ levels in IU1-treated ScCAD5 cells. This depletion of PrP^C^ could partly account for the marked reduction in PrP^Sc^ load in IU1-treated ScCAD5 cells, due to a reduction in the rate of ongoing prion conversion. In addition, proteasome activation by IU1 may facilitate increased clearance of misfolded PrP^Sc^ conformers, which we recently identified as likely substrates of the UPS [12].

The dual effect of proteasome activation on clearance of polyubiquitinated conjugates and reduction in PrP^Sc^ load is likely to reduce proteotoxic stress in prion-infected cells. Since immortalised cell lines such as the CAD5 cells used in the present study do not display prion-induced cell death, they cannot accurately model in vivo cytotoxicity observed in prion-infected mice. To establish whether UPS activation has a beneficial impact on rates of prion propagation and disease incubation time, it will therefore be necessary to test novel proteasome activator compounds in vivo. A recent study by Wilson and colleagues did not observe evidence of altered proteasome activity upon IU1 administration in wild-type mice [41]. This highlights the significant challenge faced in the development of pharmacological compounds to enhance UPS activity in vivo.

In this study, we have demonstrated that impairment of the UPS precedes the development of early behavioural symptoms and, more importantly, neuronal loss in prion-infected mice. Early intervention to compensate for accumulation of misfolded proteins by induction of protein catabolism may halt the progressive neurotoxicity observed in prion disease. In the absence of therapies to upregulate the UPS, the development of pharmacological compounds that stimulate UPS activity and are capable of crossing the blood–brain barrier remains a research priority.

## Electronic supplementary material

Supplementary material 1 (DOCX 8899 kb)
