# Supplementary material for: Prion-mediated neurodegeneration is associated with early impairment of the ubiquitin–proteasome system
Source: Acta Neuropathol. 2015 Dec 8;131:411–25. doi: 10.1007/s00401-015-1508-y (PMC4752964; doi:10.1007/s00401-015-1508-y)
Supplement: Supplementary file 1 — Supplementary material 1 (DOCX 8899 kb) [file 401_2015_1508_MOESM1_ESM.docx]

**Supplementary material**

**Table S1** Primary antibodies used for immunofluorescent staining of cryosections

| **Antibody** | **Dilution** | **Supplier** |
| --- | --- | --- |
| Anti-GFP (rabbit) | 1: 1000 | Invitrogen (A11122) |
| Anti-NeuN | 1:1000 | Millipore (MAB377) |
| Anti-GFAP | 1:1000 | Abcam (ab4674) |

**Table S2** Secondary antibodies used for immunofluorescent staining of cryosections

| **Antibody** | **Dilution** | **Supplier** |
| --- | --- | --- |
| AlexaFluor®488 Goat anti-rabbit IgG | 1: 500 | Invitrogen (A11034) |
| AlexaFluor®568 Goat anti-rabbit IgG | 1:500 | Invitrogen (A11011) |
| AlexaFluor®647 Goat anti-chicken IgG | 1:500 | Invitrogen (A21449) |
| AlexaFluor®568 Goat anti-mouse IgG1 | 1:500 | Invitrogen (A21124) |
| AlexaFluor®488 Goat anti-chicken IgG | 1:500 | Invitrogen (A11039) |

**
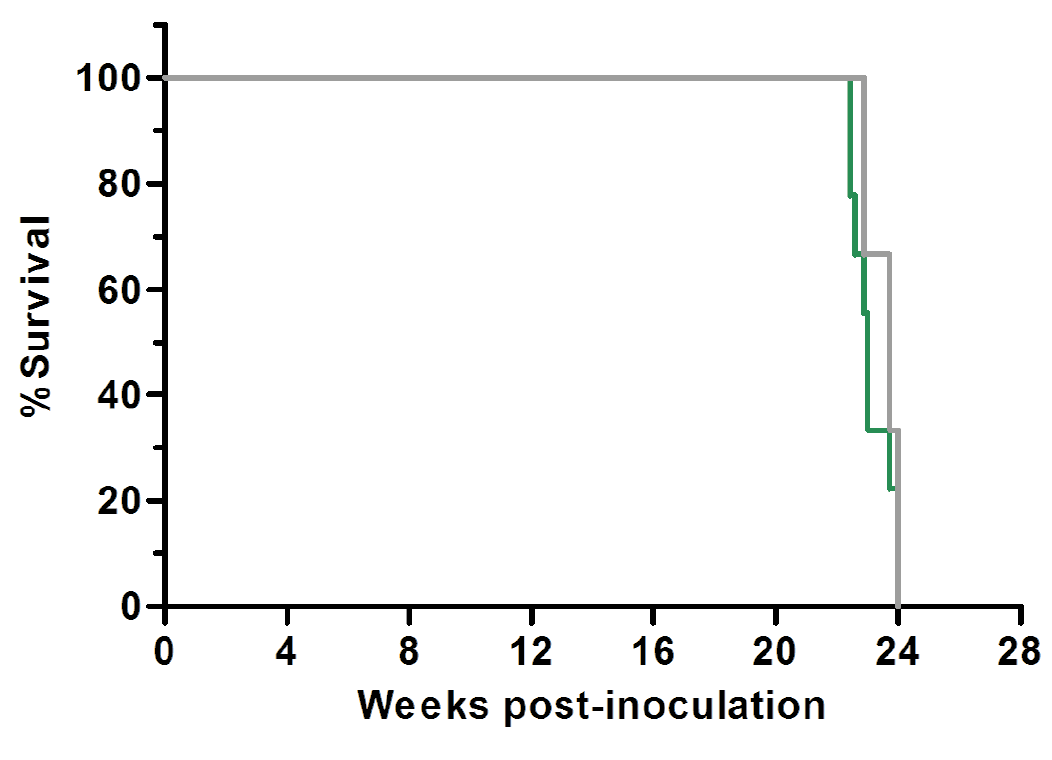
**

**Fig. S1 Prion-infected Ub^G76V^-GFP mice and wild-type littermates reach end-stage disease at 23 wpi**

Ub^G76V^-GFP mice (n=9) and wild-type littermates (n = 3) were inoculated with 30 μl of 1% RML prion-infected brain homogenate at 8-10 weeks of age. Survival curve analysis revealed mean disease incubation time of 23 wpi in Ub^G76V^-GFP mice (green line), with no significant difference compared with wild-type littermate controls (grey line) (log-rank (Mantel-Cox); p = 0.5128).


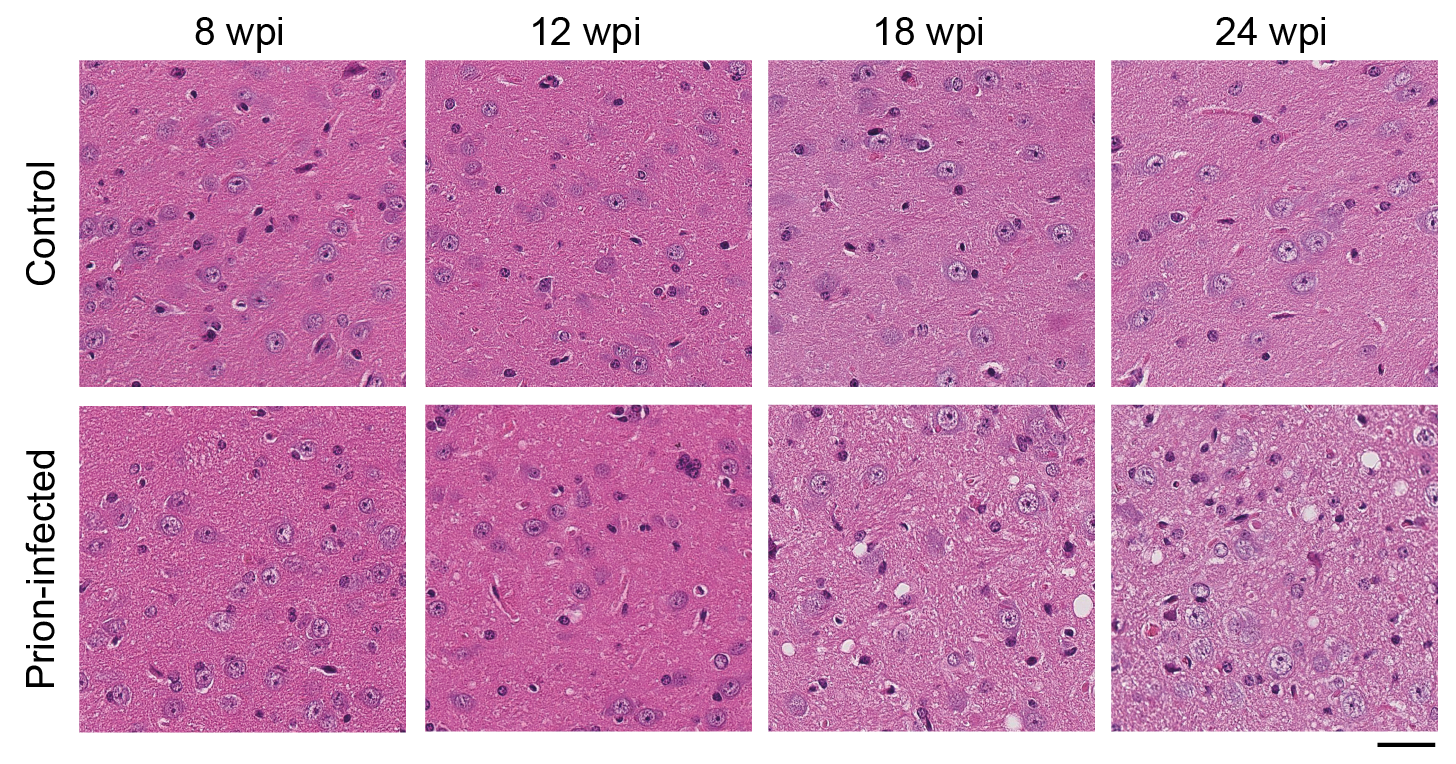


**Fig. S2 Widespread spongiosis in thalamus of prion-infected Ub^G76V^-GFP reporter mice**

Ub^G76V^-GFP reporter mice were inoculated with 30 μl of 1% RML prion-infected brain homogenate or 1% uninfected brain homogenate (control) between 8 and 10 weeks of age (n = 4 per group). Animals were culled at 8, 12, 18 and 23 wpi. H & E staining of sagittal sections reveals extensive vacuolation of neuropil in prion-infected mice at 18 wpi. Data are representative of n = 4 mice. Scale bar = 40 μm.


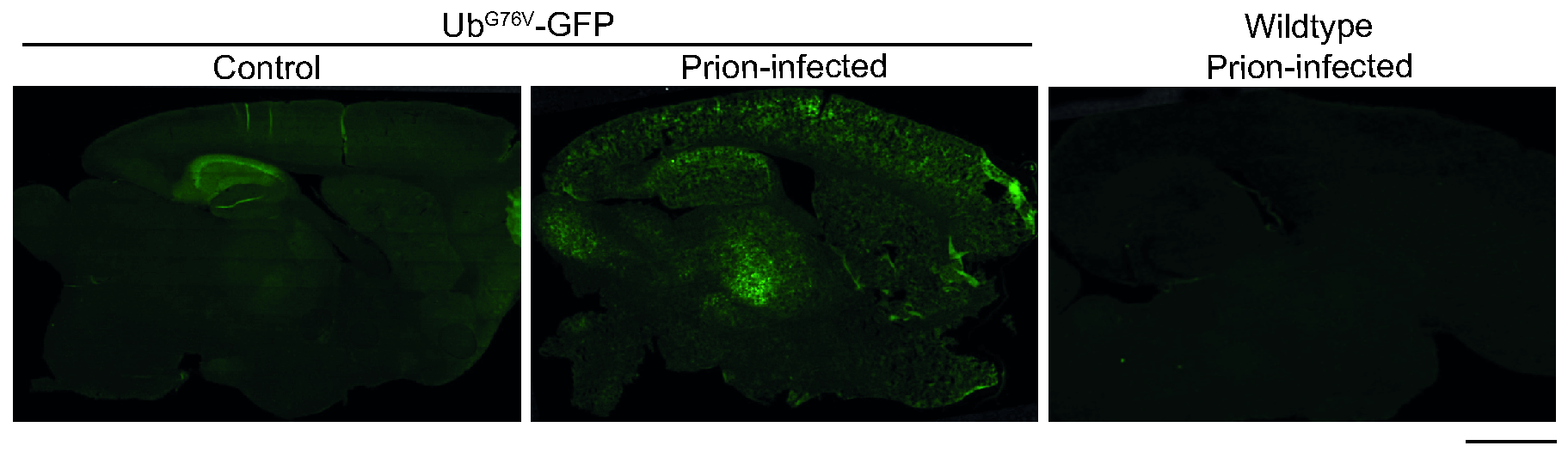


**Fig. S3 Anatomical distribution of Ub^G76V^-GFP reporter accumulation in prion-infected mice**

Ub^G76V^-GFP reporter mice were inoculated with 30 μl of 1% RML prion-infected brain homogenate or 1% uninfected brain homogenate (control) between 8 and 10 weeks of age (n = 4 per group). Animals were culled at 18 wpi. Anti-GFP immunofluorescent staining of whole sagittal sections reveals widespread accumulation of the Ub^G76V^-GFP reporter with heaviest deposition in the thalamus. Strong anti-GFP immunofluorescent staining was observed in the hippocampus of Ub^G76V^-GFP reporter mice, independent of prion infection. Data are representative of n = 4 mice. Scale bar = 2 mm.

**
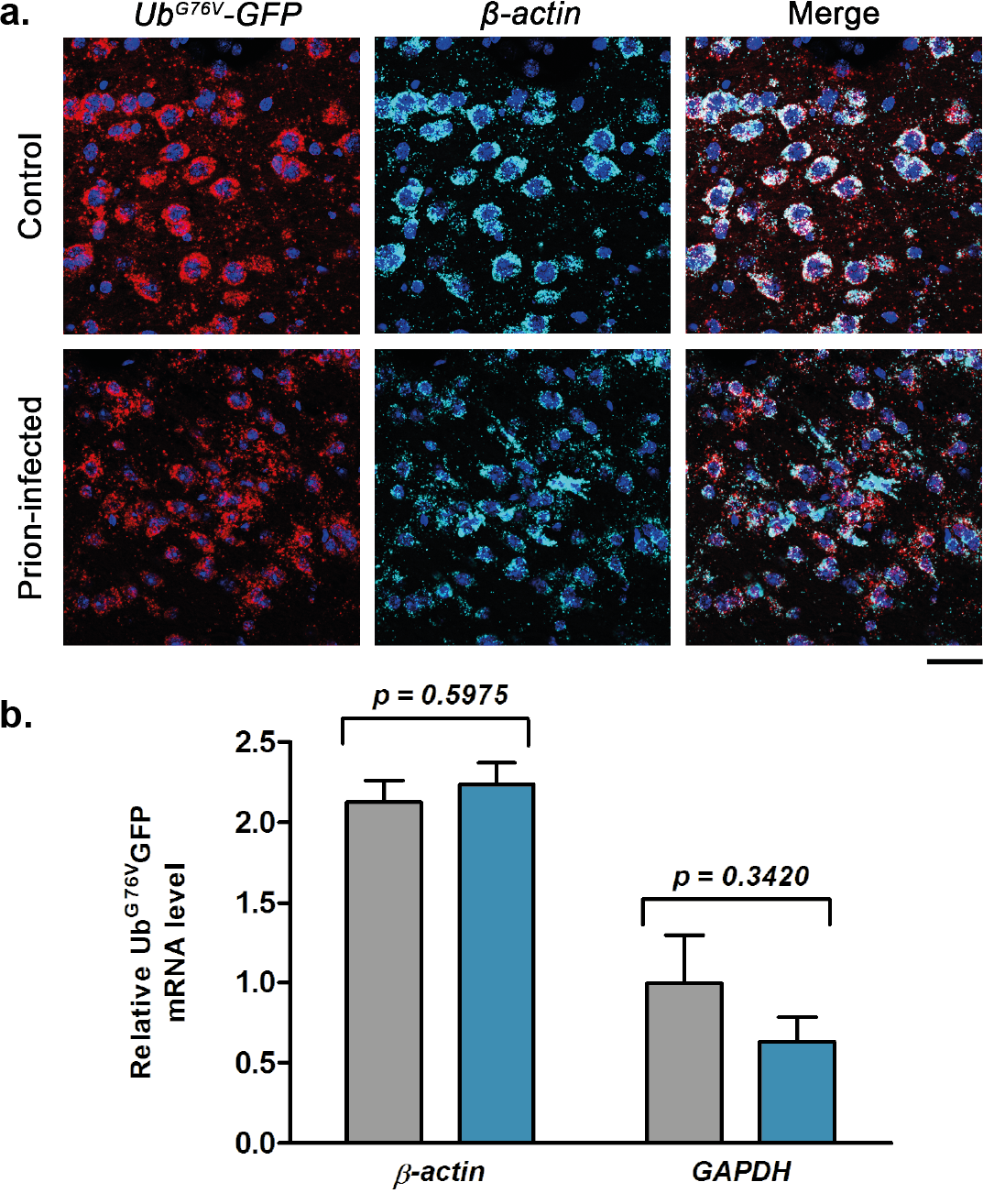
**

**Fig. S4 Ub^G76V^-GFP reporter transcript levels are not upregulated by prion infection**

**(a)** *Ub^G76V^-GFP* transcript levels were quantified in the thalamus of prion-infected and control-inoculated Ub^G76V^-GFP reporter mice at 18 wpi by RNA *in situ* hybridisation. Specific binding of the *GFP* probe was revealed by development with a fast-red substrate (red); specific binding of the *β-actin* probe was developed with a fast-blue substrate (cyan). Nuclei were stained with DAPI (blue). Scale bar = 40 μm. **(b)** Relative *Ub^G76V^-GFP* mRNA levels in prion-infected (blue bars) and control-inoculated (grey bars) Ub^G76V^-GFP reporter mice, normalised to *β-actin* or *GAPDH* housekeeping genes. Data are mean ± SEM (n = 3 per group). (P values as displayed; two-tailed Student’s t-test).


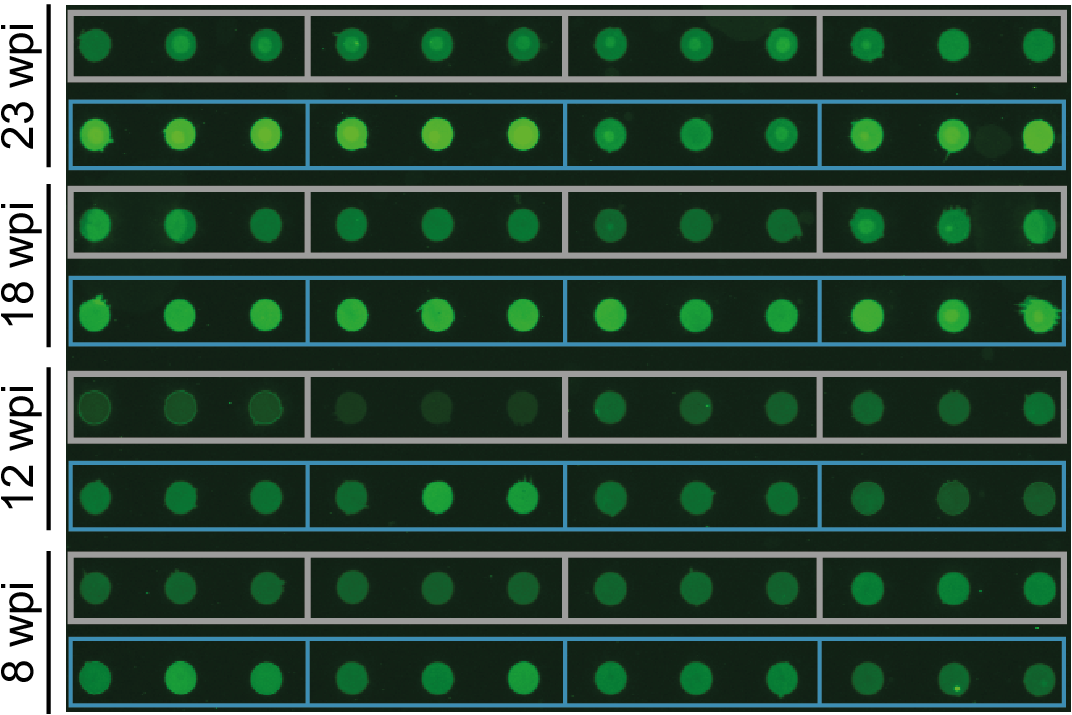


**Fig. S5 Dot blot analysis of polyubiquitinated conjugate levels in brain extracts of prion-infected and uninfected control wild-type mice**

Polyubiquitinated conjugate levels in whole brain homogenates of prion-infected (blue boxes) and uninfected control (grey boxes) mice determined by dot blot analysis. Image shows n = 4 per group per time-point, with three technical replicates for each. Quantification of the data is shown in **Fig. 6a**.


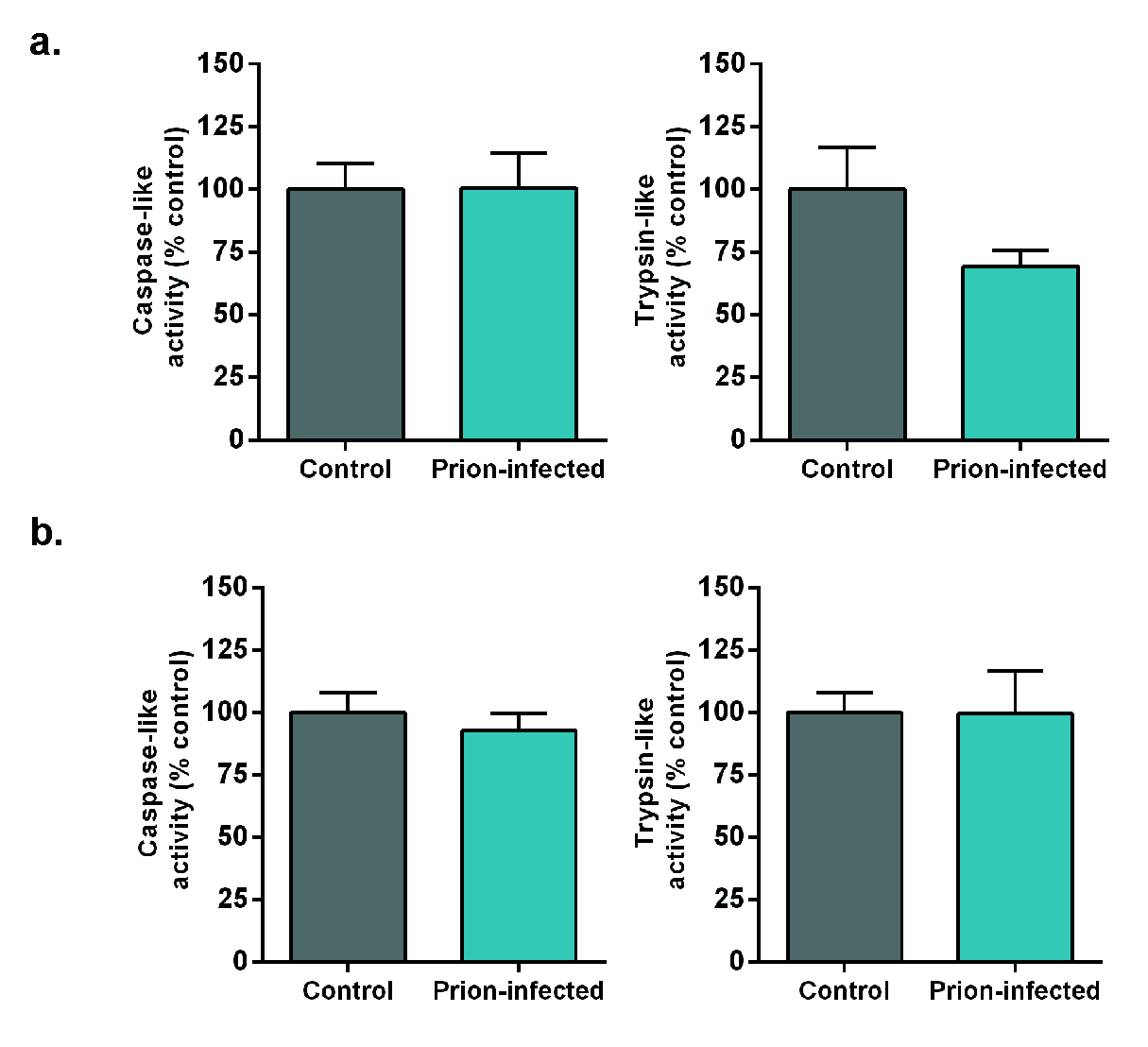


**Fig. S6 Catalytic activities of caspase- and trypsin-like sites of the 26S proteasome in prion-infected Ub^G76V^-GFP reporter mice**

Ub^G76V^-GFP reporter mice were inoculated with 30 μl of 1% RML prion-infected brain homogenate or 1% uninfected brain homogenate (control) and culled at 12 wpi **(a)** or 18 wpi **(b)**. Thalamic tissue was homogenised and caspase- and trypsin-like activity assessed by measuring fluorescence generated from cleavage of site-specific peptide substrates, adjusted to an epoxomicin-treated control. Data are percentage mean ± SEM, expressed relative to control (n = 5 per group). No significant difference in caspase- or trypsin-like activities was observed (two-tailed Student’s *t*-test).


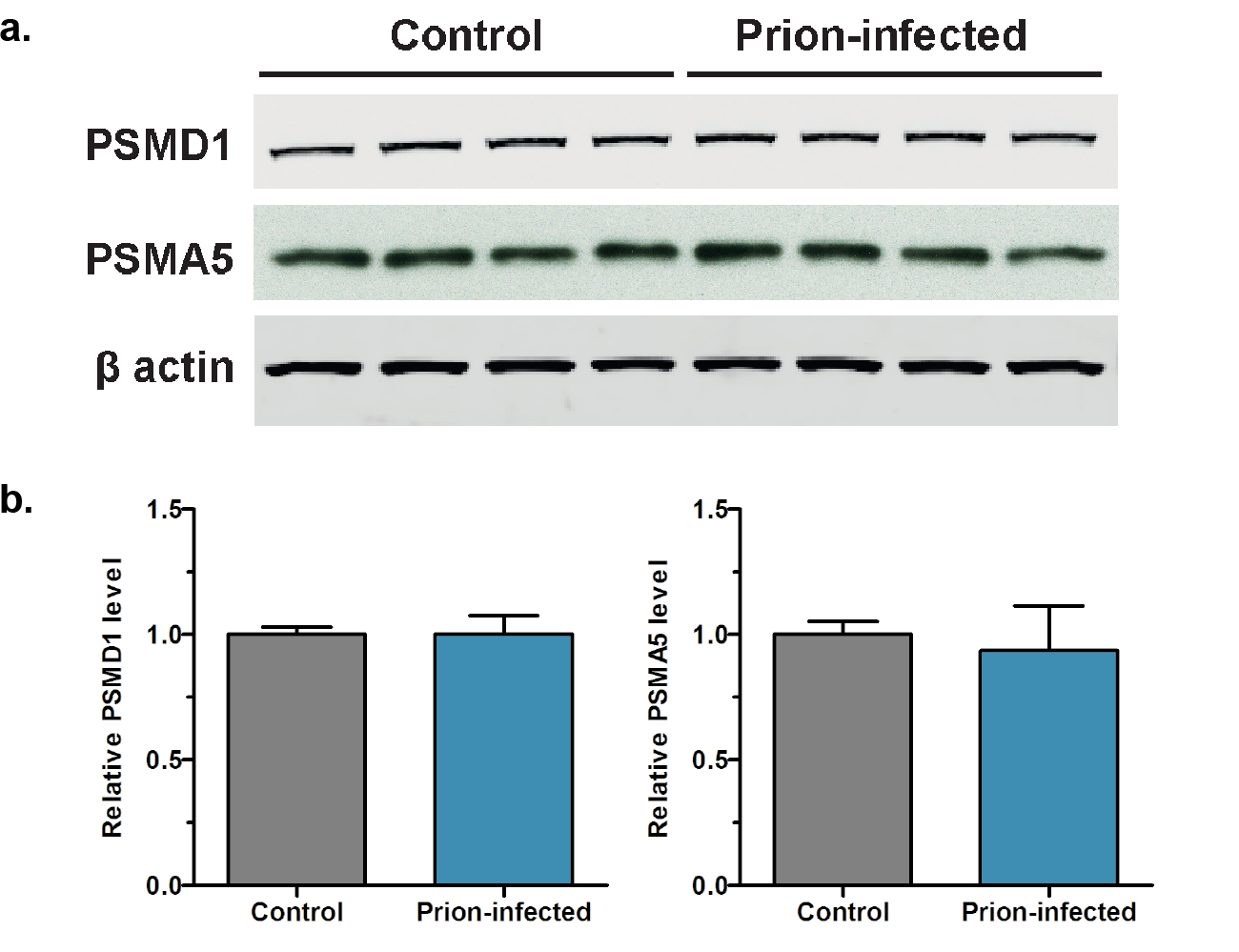


**Fig. S7 Levels of 19S and 20S proteasome subunits remain unaltered in brains of prion-infected mice**

Ub^G76V^-GFP reporter mice were inoculated with 30 μl of 1% RML prion-infected brain homogenate or 1% uninfected brain homogenate (control) and culled at 18 wpi. **(a)** Thalamic tissue was homogenised and analysed for levels of the 20S subunit PSMA5 and 19S subunit PSMD1 by Western blotting. **(b)** Quantification of immunoblots by densitometry revealed no significant change in levels of PSMD1 or PSMA5 when normalised to the β-actin housekeeping gene. Data are mean ± SEM, expressed relative to control (two-tailed Student’s t-test; n = 4 per group).
